# *Agave* as a model CAM crop system for a warming and drying world

**DOI:** 10.3389/fpls.2015.00684

**Published:** 2015-09-24

**Authors:** J. Ryan Stewart

**Affiliations:** Department of Plant and Wildlife Sciences, Brigham Young UniversityProvo, UT, USA

**Keywords:** *Agave*, agriculture, bioenergy, CAM, century plant, stress physiology, succulent

## Abstract

As climate change leads to drier and warmer conditions in semi-arid regions, growing resource-intensive C_3_ and C_4_ crops will become more challenging. Such crops will be subjected to increased frequency and intensity of drought and heat stress. However, agaves, even more than pineapple (*Ananas comosus*) and prickly pear (*Opuntia ficus-indica* and related species), typify highly productive plants that will respond favorably to global warming, both in natural and cultivated settings. With nearly 200 species spread throughout the U.S., Mexico, and Central America, agaves have evolved traits, including crassulacean acid metabolism (CAM), that allow them to survive extreme heat and drought. Agaves have been used as sources of food, beverage, and fiber by societies for hundreds of years. The varied uses of *Agave*, combined with its unique adaptations to environmental stress, warrant its consideration as a model CAM crop. Besides the damaging cycles of surplus and shortage that have long beset the tequila industry, the relatively long maturation cycle of *Agave*, its monocarpic flowering habit, and unique morphology comprise the biggest barriers to its widespread use as a crop suitable for mechanized production. Despite these challenges, agaves exhibit potential as crops since they can be grown on marginal lands, but with more resource input than is widely assumed. If these constraints can be reconciled, *Agave* shows considerable promise as an alternative source for food, alternative sweeteners, and even bioenergy. And despite the many unknowns regarding agaves, they provide a means to resolve disparities in resource availability and needs between natural and human systems in semi-arid regions.

## Introduction

As arable land continues to degrade and diminish, and as the Earth's population continues to spiral upward (Gerland et al., 2014) in a warming and drying world (Fischer et al., 2005; Howden et al., 2007; Dai, 2013; Cook et al., 2014), demand will ramp up for high-yielding crops that will be productive in semi-arid and arid regions. Few C_3_ or C_4_ crop species, however, exhibit traits that enable them to be sustainably productive in dry and hot climates, particularly in nutrient-poor soils (Pimienta-Barrios, 1994; Lobell and Field, 2007; Somerville et al., 2010; Davis et al., 2011; Challinor et al., 2014; Owen and Griffiths, 2014). Photosynthesis via the C_4_ pathway enables several crops, such as corn (*Zea mays*), sugarcane (*Saccharum officinarum*), and sorghum (*Sorghum bicolor*), to tolerate extremes in heat, but maintaining high yields depends on having reliable sources of irrigation water (Jaggard et al., 2010; Vanloocke et al., 2010; Le et al., 2011; Black et al., 2012; Zhuang et al., 2013; Goldstein et al., 2014; Yimam et al., 2014). The need for heat- and drought-tolerant crops is especially pronounced in arid and semi-arid regions, which cover nearly 40% of the world's land surface area (Ramankutty et al., 2008; Vorosmarty et al., 2010; Borland et al., 2014). In these regions, insufficient rainfall and intense evapotranspiration act as barriers to the cultivation of many economically important C_3_ and C_4_ crops (Borland et al., 2009).

Although limited in number, some crop species fix CO_2_ through an alternate form of photosynthesis, crassulacean acid metabolism (CAM), which maximizes water-use efficiency by shifting most CO_2_ uptake to the night (Borland et al., 2009; Yang et al., 2015). Cooler nighttime temperatures reduce the vapor pressure gradient between their leaves and the air, resulting in markedly lower transpiration rates as compared to C_3_ and C_4_ plants (Griffiths, 1988; Winter and Smith, 1996). Consequently, CAM confers the ability to plants to be highly water-use efficient in hot, water-limited environments. While CAM is found in several families, approximately 7% of all plant species use this photosynthetic pathway (Silvera et al., 2010). Most CAM plants are small and lack any apparent benefits as crops; CAM and allied traits, such as succulence, waxy cuticles, and low stomatal conductance, largely allow these plants to survive in semi-arid and arid lands (Szarek et al., 1973; Cushman, 2001; Matiz et al., 2013). Several CAM crops exist, such as *Aloe vera* (Xanthorrhoeaceae), *Hylocereus* spp. (Cactaceae), and *Vanilla planifolia* (Orchidaceae) (Yang et al., 2015), but several *Agave* species (Asparagaceae); pineapple (*Ananas comosus*, Bromeliaceae); and prickly pear cactus (*Opuntia ficus-indica*, Cactaceae) and other species in the genus, have been cultivated for hundreds of years for food, beverages, and fiber in hot and drought-prone regions of the world (Gentry, 1982; Griffith, 2004; Clement et al., 2010). The benefits and uses of these crops are outlined in Table 1.

**Table 1:** **Key crassulacean acid metabolism (CAM) crops and information related to their historic, current, and potential end uses and their area of cultivation**.

**Species**	**Harvested part**	**Historical and/or current uses**	**Potential end uses**	**Area of production**
*Agave angustifolia*	Leaves, stem, sap	Distilled alcoholic beverage (mezcal)^a^	Bioenergy from soluble carbohydrates and lignocellulose^b^^,^ ^c^^,^ ^d^, medicine^e^, prebiotic^e^, food additive^f^	Mexico (Oaxaca)^a^
*Agave mapisaga*	Leaves, stem, sap	Non-distilled alcoholic beverage (pulque)^a^^,^ ^g^	Bioenergy from soluble carbohydrates and lignocellulose^c^	Mexico (Mexico, Tlaxcala, Hidalgo, Queretaro, Mexico DF, Puebla, Morelos, San Luis Potosi)^a^
*Agave salmiana*	Leaves, stem, sap	Non-distilled, distilled alcoholic beverage (pulque, mezcal)^a^^,^ ^g^, sweetener (syrup)^h^^,^ ^i^	Bioenergy from soluble carbohydrates and lignocellulose^b^^,^ ^j^, medicine^e^	Mexico (San Luis Potosi)^a^
*Agave tequilana*	Leaves, stem, sap	Distilled alcoholic beverage (tequila)^a^^,^ ^k^, sweetener (syrup)^h^^,^ ^i^^,^ ^l^	Bioenergy from soluble carbohydrates and lignocellulose^b^^,^ ^c^^,^ ^d^^,^ ^j^^,^ ^m^, prebiotic^e^	Mexico (Jalisco, Guanajuato, Michoacan, Nayarit, Tamaulipas)^n^
*Agave fourcroydes*	Leaves	Fiber^o^^,^ ^p^, distilled alcoholic beverage^q^	Bioenergy from soluble carbohydrates and lignocellulose^r^^,^ ^s^, leaf fiber as reinforcement agent for composite fibers^t^^,^ ^u^	Mexico (Yucatan)^v^
*Agave lechuguilla*	Leaves	Fiber^w^^,^ ^x^	Leaf fiber as reinforcement agent for composite fibers^y^, medicine^z^^,^ ^aa^^,^ ^bb^	Mexico (Coahuila, Chihuahua, Nuevo Leon, Durango, San Luis Potosi, and Zacatecas)^cc^
*Agave sisalana*	Leaves	Fiber^dd^^,^ ^ee^	Bioenergy from soluble carbohydrates and lignocellulose^s^, leaf fiber as reinforcement agent for composite fibers^ff^, medicine^gg^, bioinsecticide^gg^	Brazil, Kenya, Tanzania^s^^,^ ^hh^
*Ananas comosus*	Fruit, leaves	Fruit, beverage^ii^	Bioenergy from soluble carbohydrates^jj^, leaf fiber as reinforcement agent for composite fibers^kk^, medicine^ll^, food additive^mm^	Brazil, Costa Rica, Phillipines, Thailand^ii^
*Opuntia ficus-indica*	Fruit, cladodes	Fruit^nn^, nopales^oo^, forage^pp^, cochineal dye^qq^	Bioenergy from soluble carbohydrates and lignocellulose^rr^, antioxidants^ss^^,^ ^tt^^,^ ^aa^, medicine^uu^^,^ ^vv^^,^ ^ww^, dyes^xx^	Algeria, Brazil, Chile, Mexico, Sicily^yy^

a *Lappe-Oliveras et al., 2008*;

b *Escamilla-Trevino, 2012*;

c *Garcia-Moya et al., 2011*;

d *Núñez et al., 2011*;

e *Santos-Zea et al., 2012*;

f *Rivera et al., 2010*;

g *Ortiz-Basurto et al., 2008*;

h *Willems and Low, 2012*;

i *Garcia-Pedraza et al., 2009*;

j *Davis et al., 2011*;

k *Cedeño Cruz, 2003*;

l *Montanez Soto et al., 2011*;

m *Owen and Griffiths, 2014*;

n *Zizumbo-Villarreal and Colunga-GarcíaMarín, 2008*;

o *Colunga-GarcíaMarín, 2003*;

p *Nobel, 1985*;

q *Rendon-Salcido et al., 2009*;

r *Martinez-Torres et al., 2011*;

s *Davis and Long, 2015*;

t *Gonzalez-Murillo and Ansell, 2009*;

u *May-Pat et al., 2013*;

v *García de Fuentes and Morales, 2000*;

w *Bautista Lopez and Martinez Cruz, 2012*;

x *Pando-Moreno et al., 2008*;

y *Velasquez-Martinez et al., 2011*;

z *Mendez et al., 2012*;

aa *Santos-Zea et al., 2012*;

bb *Ramos Casillas et al., 2012*;

cc *Pando-Moreno et al., 2004*;

dd *Colunga-GarciaMarin and May-Pat, 1993*;

ee *Lock, 1962*;

ff *Barreto et al., 2011*;

gg *Ribeiro et al., 2013*;

hh *Kimaro et al., 1994*;

ii *Carr, 2012*;

jj *Wang et al., 2006*;

kk *Mohanty et al., 2000*;

ll *Gupta et al., 2014*;

mm *Arshad et al., 2014*;

nn *Basile, 2001*;

oo *Saenz-Hernandez et al., 2002*;

pp *Nefzaoui and Ben Salem, 2002*;

qq *Ervin, 2012*;

rr *Kuloyo et al., 2014*;

ss *Butera et al., 2002*;

tt *Benayad et al., 2014*;

uu *Wiese et al., 2004*;

vv *Kuti, 2004*;

ww *Saleem et al., 2006*;

xx *Chavez-Moreno et al., 2009*;

yy *Griffith, 2004*.

Given the dearth of efforts to develop and improve CAM species as crops, priority needs to be given to research on species that will yield the greatest impact in addressing human needs. On a global scale, *Ananas comosus* ranks as the most commercially important CAM crop (Py et al., 1987; Carr, 2012; Yang et al., 2015). Besides being mainly grown for its fresh fruit in different parts of the world (Table 1), other uses of *A. comosus* include alcohol (Araujo et al., 2009), additive agents (Ha et al., 2012), and fiber (Luo and Netravali, 1999). However, its physiology limits its cultivation to arable lowlands in subtropical or tropical areas (Carr, 2012), which only constitute 5% of the earth's total land surface (Ramankutty et al., 2008). In addition, high levels of irrigation and fertilizer are required to sustain high yields of *A. comosus* (Obiefuna et al., 1987; Alvarez et al., 1993; Carr, 2012). And while leaves can be used as a source of fiber or bioenergy (Moya and Camacho, 2014; Zainuddin et al., 2014), such applications will likely be only regional in scale and application given that *A. comosus* can only be grown in tropical regions. Moreover, climate projection models suggest that while climate change will expand suitable cropland in northern latitudes, tropical regions could become less suitable for agriculture (Notaro et al., 2012; Zabel et al., 2014).

Compared with *A. comosus, O. ficus-indica* and related species offer more versatility as a crop due to their high productivity (Acevedo et al., 1983; Nobel et al., 1992), ability to be grown in semi-arid lands (Nobel, 1988), and diverse end uses (Mohamed-Yasseen et al., 1996; Soberon et al., 2001; Sáenz et al., 2013). Largely grown in areas in Mexico, Brazil, and other parts of the world with mild to warm winters, *O. ficus-indica* can be cultivated for its sweet or sour fruit, young cladodes as vegetables for human and livestock consumption, and as a host plant for cochineal insects (*Dactylopius coccus*) for the production of valuable dyes (Table 1). Besides the risks associated with widespread planting of clonal monocultures (Ploetz, 1994; Helmers et al., 2001; Siebert, 2002; Knoke et al., 2005), the sensitivity of *O. ficus-indica* to frost limits its production to areas absent of chilling to subfreezing temperatures (Nobel, 2010). Other highly productive *Opuntia* species, which could be considered as crops, exhibit more cold hardiness than *O. ficus-indica*, but their prolific spines and glochids preclude their widespread use. In addition, *O. ficus-indica* and other members of the genus have been found to be highly invasive in some semi-arid environments (Van Sittert, 2002; Shackleton et al., 2007; Bartomeus et al., 2008; Osmond et al., 2008). These limitations of *O. ficus-indica* underscore the challenges in using it as a model CAM crop system.

*Agave*, more so than *A. comosus* and *O. ficus-indica*, offers the greatest potential to be more extensively used as a crop in a warming and drying world (Yang et al., 2015). In the southwestern U.S., Mexico, and Central America, several pre-Columbian indigenous groups obtained food, beverage, fiber, medicine, and other vital products from several species within the *Agave* genus to persist despite living under harsh environmental conditions (Castetter and Underhill, 1935; Castetter et al., 1938; Krochmal et al., 1954; Brugge, 1965; Minnis and Plog, 1976; Parsons and Parsons, 1990; Parsons and Darling, 2000; Allison et al., 2008; Anderies et al., 2008; Fish and Fish, 2014). Table 1 provides an overview of the primary products derived from the more widely cultivated *Agave* species. The *Agave* genus constitutes a large and diverse group of stress-tolerant succulents native to semi-arid regions of the Nearctic and Neotropics (Gentry, 1972, 1978, 1982; Garcia-Mendoza, 2002, 2007).

Modern society, particularly populated areas where water is severely limited, could benefit from the utilization of agaves as crops. The high productivity of several *Agave* species, coupled with their use of the CAM pathway, have led some to believe they can be used to resolve pressing environmental issues and energy needs (Borland et al., 2009; Yan et al., 2011; Yang et al., 2015). The genus also has the potential to serve as a model to determine how drought-tolerant crops could help resolve disparities between increasingly scarce resources and pressing societal needs, particularly in connecting traditional crop systems with modern agricultural approaches (Dubé et al., 2014). This is especially the case for semi-arid regions that are prone to prolonged periods of drought, such as the southwestern U.S. (Gleick, 2010; Sabo et al., 2010; Dai, 2011). Agricultural activities consume nearly 80% of available water in the Southwest (MacDonald, 2010), which will likely increase due to the needs associated with growing urban and suburban populations (Georgescu et al., 2013). Demand will continue to spiral upward for the reallocation of water from agricultural and industrial operations to meet domestic needs of large population centers in these semi-arid regions (Hurd et al., 1999; Vörösmarty et al., 2000; Tanaka et al., 2006; Gleick, 2010; MacDonald, 2010). This will only aggravate the situation for farmers and producers who not only have to deal with drought-associated reductions in available water, but also challenges due to increased domestic consumption (Gober and Kirkwood, 2010; Larson et al., 2013; Lobell et al., 2014). The margin of error for cultivating conventional, high-water-use crops will increasingly become thinner, which could ultimately lead to limited food choice for consumers as well as increased compromises in food security (Gleick, 2010; MacDonald, 2010). These risks underscore the need to focus on innovative solutions (Gleick, 2010), such as the widespread cultivation of crops in areas that could be subject to widespread, severe droughts (Cook et al., 2015). Evaluating *Agave* as a model crop system will be a promising step in that direction.

Notwithstanding the potential benefits of using *Agave* as a model crop system, little has been done to synthesize what is known concerning its more traditional, but still modern, uses as food, beverage, and fiber, in line with its more contemporary and innovative uses in the sweetener (Willems and Low, 2012; Zamora-Gasga et al., 2014) and bioenergy industries (Davis et al., 2011; Holtum et al., 2011; Yan et al., 2011; Escamilla-Trevino, 2012). The integrative synthesis of information in this review provides a strong platform on which to further the characterization of *Agave* as a model CAM crop.

### Why agave?

Over the past several million years, agaves evolved physiological mechanisms and anatomical traits, which enable them to be productive despite growing in harsh semi-arid environments, where water and nutrients are severely limited (Nobel, 1988, 1994; Good-Avila et al., 2006; Arakaki et al., 2011). The CAM pathway enables agaves to colonize semi-arid environments where water is scarce and soil surface temperatures often exceed 55°C (Nobel, 1994). Moreover, the succulent and fiber-rich nature of agave leaves allows for the continuation of CO_2_ fixation and other vital biochemical reactions during extended periods of drought, which can last up to 7 years or more (Lüttge, 2004; Garcia-Mendoza, 2007; Matiz et al., 2013). In addition, the rosette arrangement of agave leaves allows for maximal absorption of photosynthetically active radiation (Nobel, 1994), and allows for the funneling of water to their relatively shallow root systems (Gentry, 1982; Matiz et al., 2013). The roots of agaves can shrink in response to drying soils, minimizing water loss (Nobel and Sanderson, 1984), but they can also quickly generate fine roots to permit rapid water uptake after a short-lived rain event (North and Nobel, 1998). Moreover, dead leaves accumulate at the base of agaves, buffering living leaf tissue from high soil surface temperatures, which can range from 50 to 55°C (Nobel, 1994). Dead agave leaves also likely contribute to reduced soil evaporation and increased soil organic matter.

Agaves have several other traits that have made them suitable as crops over several millennia. These include their starch-rich stems (caudices) (Hodgson, 2001), semelparous flowering (Eguiarte et al., 2000), long-lived perennial habit (Gentry, 1982; Nobel, 1994; Bowers et al., 1995), natural proclivity to grow on rocky, infertile soils (Shreve, 1942; Gentry, 1982; Nobel, 1988, 1989, 2010), fiber-rich leaves (Garcia-Mendoza, 2007), and ease of vegetative propagation (Gentry, 1972; Arizaga and Ezcurra, 1995).

Due to such traits, global warming could lead several agaves to naturally expand their ranges northward into the Nearctic (Lotze-Campen and Schellnhuber, 2009; Lewis et al., 2015), and to also be cultivated over a much wider area than is currently possible (Nobel, 1996, 2010). Consequently, agaves have the potential to play a more prominent role in natural and human systems in semi-arid lands in North America and other parts of the world. Society will increasingly need such plants to sustain food and energy demands associated with growing populations (Godfray et al., 2010; Foley et al., 2011; Tilman et al., 2011).

### Agave as food

Of the approximately 200 species in the *Agave* genus, several taxa have long benefited indigenous groups throughout the U.S. Southwest, Mexico, and Central America as food (Castetter and Underhill, 1935; Castetter et al., 1938; Allison et al., 2008; Anderies et al., 2008). Indeed, cultivation of agaves as food crops was widespread in the southwestern U.S. and Mexico (Minnis and Plog, 1976; Evans, 1990; Fish and Fish, 1992), which was largely due to its unique morphology and physiology. Prior to the emergence of the inflorescence, the head of the plant contains large amounts of carbohydrates (Hodgson, 2001), which is used to fuel the elongation of the inflorescence. Harvesting of agave heads, which primarily occurred in the early spring, was considered by many tribal groups to be optimal prior to elongation of the inflorescence, which was manifest by morphological changes in the leaves, often several months before bolting occurred (Arizaga and Ezcurra, 1995; Hodgson, 2001). Through the energy gained from heads and inflorescences, agaves provided subsistence for tribal groups in the southwestern U.S. and Mexico to survive under harsh conditions (Callen, 1965; Hodgson, 2001; Colunga-GarcíaMarín and Zizumbo-Villarreal, 2007). Cultivation of agaves possibly helped to support large settlements in semi-arid northern Mexico between AD 500 and 900 (Anderies et al., 2008). In addition, the adaptability of agaves to grow in mesic and dry soils helped support the emergence of large cities in the resource-limited highlands of central Mexico (Parsons and Darling, 2000). Indeed, in pre-Columbian times, several indigenous peoples foraged or cultivated agaves for food (Minnis and Plog, 1976; Fish et al., 1985; Trombold, 1985; Evans, 1990; Fish and Fish, 1992; Hodgson and Slauson, 1995; Adams and Adams, 1998; Phippen, 1999).

Evidence of agave cultivation includes tabular stone knives (used to remove leaves from agave heads) (Parsons and Parsons, 1990), rock piles, and roasting pits, all of which have agave phytolith residues present as the tell-tale signature of agave cultivation and consumption (Cummings and Puseman, 1994; Reinhard and Danielson, 2005; Fish and Fish, 2014). While long a primary food source for peoples in Central America, agave was partially displaced by the widespread cultivation of maize, beans, and squash, and became a crop that was only used in times of scarcity (Turkon, 2004; Colunga-GarcíaMarín and Zizumbo-Villarreal, 2007).

Unlike most crops that indigenous peoples cultivated, agaves persist without human input due to their succulent leaves, low water use, and ease of establishment (Adams and Adams, 1998). Moreover, agave heads and vegetative offsets could be transported long distances by pre-Columbian indigenous groups (Castetter et al., 1938; Hodgson et al., 1989; Radding, 2012) without the need to protect them from desiccation or mechanical damage.

Despite being a ready source of subsistence, though, agaves were not an ideal crop. Their slow growth and the amount of time it took for plants to mature (>10 years) suggests that they may have been a food source primarily during drought years (Anderies et al., 2008). Harvesting and cultivation practices varied, though, among tribes. Some groups would travel long distances to harvest agaves and then return to roast them in earth ovens (Curtis, 1907; Buckelew and Dennis, 1925; Reagan, 1929; Dering, 1999).

Several tribes spent time in the fall, winter, and spring searching for and harvesting agaves, which were in the incipient stages of flowering (Castetter et al., 1938; Hodgson, 2001). Interestingly, what were long considered to be naturally established agave populations in southern Arizona are now considered to be plants established for agricultural purposes (Fish et al., 1985; Fish and Fish, 1992, 2014; Parker et al., 2010). Agaves were cultivated by the Hohokam people in large agricultural fields associated with rock piles, terraces, and check dams (Fish et al., 1985; Leach, 2007). Fish et al. (1985) estimated that nearly 10,000 agaves were annually harvested from a standing crop of more than 100,000 plants. *Agave murpheyi*, which was cultivated by the Hohokam, has several traits indicating it was domesticated as a crop (Adams and Adams, 1998). Unlike many agaves, the time to flowering for *A. murpheyi* is relatively short (~9 years). In addition, the leaves of *A. murpheyi* are not as caustic as other agaves, which in addition to their small marginal teeth makes them relatively easy to harvest. Also, the species flowers in midwinter, which is when the heads are ready to be harvested, and is when very little food would have been available to the local tribes. Most agaves in the region flower 2–3 months after *A. murpheyi*.

After harvesting and transporting the starch-filled heads, most tribes would prepare them by roasting them for 40 h or longer in rock-lined earth ovens (Callen, 1965; Hodgson, 2001; Allison et al., 2008; Leach and Sobolik, 2010; Rousso, 2010; Radding, 2012). The material was then eaten immediately or pounded into sheets, dried, and then stored for later consumption or trade with neighboring tribes (Fish et al., 1985; Dering, 1999). Sobolik (1996) suggested, however, that winter consumption of roasted heads of *A. lechuguilla* by indigenous peoples in southern Texas and northern Mexico may have induced some short-term nutritional stress. Although the plants provide carbohydrates, they lack a sufficient diversity of amino acids, trace elements, and minerals necessary for sustaining good health (Sobolik, 1996). In addition, they contain a high proportion of non-digestible carbohydrates, in the form of inulin, which reduces their net energy value (Leach, 2007). To help meet caloric requirements, agaves were likely eaten with other crops, such as maize (Leach, 2007; Anderies et al., 2008). Such a mixed diet also helped prevent nutritional imbalances (Anderies et al., 2008). Agaves were an important source of carbohydrates for several of these tribes (Castetter and Underhill, 1935; Castetter et al., 1938; Bruman, 1941; Brugge, 1965; Felger and Moser, 1970; Bennett and Zingg, 1976; Minnis and Plog, 1976; Fish et al., 1985; Fish and Fish, 1992; Burwell, 1995; Zizumbo-Villarreal and Colunga-GarcíaMarín, 2008; Radding, 2012).

Even after the widespread cultivation of maize, the importance of agave as a crop became more prominent when the maize crop failed in drought years (Sauer, 1963; Parsons and Parsons, 1990; Anderies et al., 2008). Nelson (1992) reported that indigenous groups planted agave when maize was in short supply.

In the face of warming and drying climates, consideration should be given to cultivating agaves for modern consumption due to their ability to be grown on marginal lands in semi-arid climates with little input (Nobel, 2010). Other orphaned indigenous crops, such as quinoa (*Chenopodium quinoa*) and amaranth (*Amaranthus* spp.), have been cultivated and marketed with some degree of success (Teutonico and Knorr, 1985; Jacobsen, 2003; Rivera et al., 2010).

The slow growth of agaves plus their monocarpic flowering and vegetative reproduction pose some challenges to not only establishing and harvesting them with conventional agricultural implements, but also to improving them through classical and molecular plant breeding. And although some agaves have been reported to have impressive yields (Nobel, 1991), only a fraction of the yield harvested can be consumed and digested (Leach, 2007). Leach (2007) estimated that only 24 people would have their annual caloric requirements met from an annual harvest of nearly 10,000 agave plants, which is largely due to the high levels of non-digestible carbohydrates in the form of inulin-type fructans. Inulin-type fructans is the major carbohydrate found in agaves (Lopez et al., 2003; Mancilla-Margalli and López, 2006). However, if eaten in a mixed, balanced diet, agaves could act as an alternative fiber source (Rivera et al., 2010). They could also be used as prebiotics (Leach, 2007; Leach and Sobolik, 2010). Enzymes in digestive tracts cannot hydrolyze beta-glucosidic bonds in inulin-fructans, which consequently promotes the growth of beneficial microorganisms in mammalian intestines. Such microbial growth can lead to numerous health benefits, including reduced gut infections, improved lipid metabolism, higher mineral absorption, and reduced risk of cancer (Van Loo, 2005). Based on prehistoric diet analyses, Leach and Sobolik (2010) surmised that modern-day diets could adapt to higher levels of inulin-type fructans. Such compounds have been found to be effective in weight control in laboratory animals (Rivera et al., 2010).

The exploration of other end products of *Agave* is also warranted, including incorporation into dairy, bread, and candy products (Rivera et al., 2010). If pursued as a food crop, selections could be made for agave clones with higher levels of digestible carbohydrates. On the other hand, the high levels of inulin-fructans could possibly lead to delayed digestion in the intestinal tract, which could be beneficial for diabetics. Regardless, sensory analysis will be required to determine consumer acceptability of agave. While the likelihood of agave being widely eaten in the U.S. or other developed nations is low, opportunities exist for it to become a part of regional niche food markets.

#### Agave as beverages

Besides being used as a food crop, some agaves were also widely harvested in the wild and cultivated to produce non-alcoholic and alcoholic beverages (Gentry, 1982; Bruman, 2000; Jennings et al., 2005). In pre-Columbian Mexico, native peoples in the region would cut or remove the nascent inflorescence from reproductively mature agaves to allow the sugar-rich sap from the head to accumulate in the hollowed-out circular center (Bruman, 1941; Parsons and Parsons, 1990; Jennings et al., 2005). Agaves commonly used for beverages are collectively known in Spanish as maguey pulquero, and include *Agave americana, Agave angustifolia, Agave atrovirens, Agave ferox, Agave mapisaga, Agave salmiana*, and other species of local importance (Jennings et al., 2005; Lappe-Oliveras et al., 2008; Ortiz-Basurto et al., 2008). The sap, which was intended for fueling development of the emerging inflorescence, is called aguamiel. Aguamiel is crystalline and high in sugar content (12°Brix), with an average pH of 7.5 (Gómez-Aldapa et al., 2011). Although diminished nowadays in cultural importance, aguamiel is still harvested in central Mexico with methods used prior to Spanish colonization (Jennings et al., 2005). Aguamiel is collected at least every 12 h after scraping the hollowed area in the head (Gómez-Aldapa et al., 2011). Production lasts, on average, from 4 to 6 months. According to Nobel (2010), a single agave plant can produce 700 L of aguamiel. In recent years, there have been efforts to promote its use as an alternative traditional beverage due to its purported nutritive benefits, including fructo-oligosaccharides, which have prebiotic properties, and several essential amino acids, vitamins, and minerals (Ortiz-Basurto et al., 2008; Rivera et al., 2010).

Perhaps more culturally significant than aguamiel to indigenous groups in central Mexico was its fermented product, pulque. Pulque is a milky fermented liquid, largely made from *A. salmiana, A. mapisaga*, and *A. americana* sap (Table 1), which has an herbal aroma, low alcohol content (4–6%), and high acidity (pH = 3.5–4.2) (Jennings et al., 2005; Gómez-Aldapa et al., 2011). The drink, whose origins date back to the Early Classic period (AD 150–650), was used in religious rituals to honor the Aztec goddess, Mayahuel, and was regularly consumed by Aztec nobility (Jennings et al., 2005; Radding, 2012). Possibly more importantly, however, the beverage provided a source of calories and nutrients during times of shortfall in corn and bean production (Correa-Ascencio et al., 2014). The beverage is still consumed during festivals and significant cultural events, and is considered an important component of maternal diets in the highlands of central Mexico, due to it being rich in micronutrients (Kuhnlein, 2004). As with aguamiel, pulque is still produced using the same methods developed hundreds of years ago. After collection, aguamiel is placed in wood barrels or goatskin bags, and then transferred to larger barrels to allow for fermentation (Escalante et al., 2004). Fermentation starts within the plant due to naturally occurring microorganisms, including yeasts, lactic-acid bacteria, ethanol-producing bacteria, and exopolysaccharide-producing bacteria, but is accelerated by the addition of previously produced pulque (Escalante et al., 2004; Gómez-Aldapa et al., 2011). The entire fermentation process, which is done primarily by regional artisans under non-aseptic conditions, ranges from 12 to 48 h (Escalante et al., 2004; Gómez-Aldapa et al., 2011).

Upon fermentation, pulque is stored without preservatives in wood barrels, and is distributed daily for sale (Escalante et al., 2004). Consumption of pulque must occur within a few days after fermentation because it otherwise quickly undergoes decomposition and acquires a putrid stench (Jennings et al., 2005). Up to the first half of the twentieth century, pulque production was a profitable industry, but due to various factors, its economic importance has diminished (Ortiz-Basurto et al., 2008). However, there are currently nearly 20,000 hectares in Mexico devoted to aguamiel and pulque production, with about 200 million L produced annually (Nobel, 2010). Some companies have industrialized the process (Gómez-Aldapa et al., 2011), which could lead to increased production. However, due to inadequate hygiene conditions and the risk of contamination by pathogenic bacteria, concern exists regarding the safety related to the fermentation process of pulque production (Gómez-Aldapa et al., 2011). Gómez-Aldapa et al. (2011) found that there was low risk of consumer exposure to common pathogenic bacteria, such as *Salmonella, Staphylococcus aureus, Listeria monocytogenes, Shigella flexneri*, and *Shigella sonnei*, when pulque was processed following conventional methods. This was possibly due to the low pH of pulque and the presence of ethanol and saponins (Rivera et al., 2010; Gómez-Aldapa et al., 2011). However, *Escherichia coli* O157:H7, a virulent foodborne pathogen, was found to survive fermentation cycles in pulque production (Gómez-Aldapa et al., 2012). Before widespread production occurs, improvements in proper handling and hygiene of those producing pulque are needed to reduce consumer health risk.

Based on archaeological evidence and historical records, it has been suggested that the initial production of agave spirits, otherwise generically known as mezcal (from the Nahuatl words “metl” = agave and “ixcalli” = cooked or baked), originated in the foothills of the Colima volcanoes in the early 1600s (Walton, 1977; Zizumbo-Villarreal and Colunga-GarcíaMarín, 2008). After removal of the outer leaves, agave heads and leaf bases were harvested, cut into sections, and steam-cooked for several days to convert the starch to sugar (Gentry, 1982; Núñez et al., 2011). They were then crushed and mashed to separate the sugar-rich pulp from the bagasse. The pulp and juice was then fermented and distilled to produce various types of agave spirits. The resultant alcohol was then distilled to produce liquor. These traditional techniques are still in wide use (Valenzuela Zapata and Nabhan, 2003; Zizumbo-Villarreal and Colunga-GarcíaMarín, 2008). According to Valenzuela Zapata and Nabhan (2003), distillation allowed agave to withstand the demise many New World crops suffered with the onset of the Columbian Exchange.

Historical evidence suggests that *A. angustifolia* was the primary species used for mezcal production (Table 1), which is evident in the several cultivars of this species currently found in the region, particularly Colima and southern Jalisco (Colunga-GarcíaMarín and Zizumbo-Villarreal, 2007; Zizumbo-Villarreal and Colunga-GarcíaMarín, 2008). The native distribution of *A. angustifolia*, which is found in areas with rugged terrain and along ravines, likely favored illicit mezcal production since they were far removed from the circle of prohibition enforcement (Zizumbo-Villarreal and Colunga-GarcíaMarín, 2008). Long-established trade routes also likely fueled demand for agave spirits in mining districts north of Colima. In addition, a portable form of distillation technology imported from the Philippines possibly facilitated the spread of mezcal distillation throughout western Mexico and what is now the southwestern U.S. with agaves native to remote mountainous areas (Walton, 1977; Zizumbo-Villarreal and Colunga-GarcíaMarín, 2008).

In contrast, other authors suggest that mezcal originated in northern Jalisco in the Tequila Valley through the introduction of the Arab-type still by the Spanish (Valenzuela Zapata and Nabhan, 2003; Zizumbo-Villarreal and Colunga-GarcíaMarín, 2008), which was originally introduced for sugar cane distillation (Colunga-GarcíaMarín and Zizumbo-Villarreal, 2007). The emergence of mezcal in Mexico was likely a fusion of both distillation technologies (Bruman, 1941; Colunga-GarcíaMarín and Zizumbo-Villarreal, 2007). Moreover, recent findings suggest that rudimentary distillation techniques were developed in Colima centuries before Spanish colonization (Zizumbo-Villarreal et al., 2009).

Regardless of how agave spirits originated, the high species diversity in Colima and southern Jalisco likely played a strong role in the development of mezcal and the subsequent diffusion of its production throughout much of Mexico and the Southwest (Zizumbo-Villarreal and Colunga-GarcíaMarín, 2008). More than 40 different agave species are harvested from the wild or cultivated to produce various types of mezcal in 26 states throughout Mexico (Colunga-GarcíaMarín and Zizumbo-Villarreal, 2007; Zizumbo-Villarreal and Colunga-GarcíaMarín, 2008). Many of these mezcal variants are defined by the agaves they are derived from, but also by regional variations in the cooking, fermentation, and distillation processes. Some of the more recognized types include bacanora, raicilla, and the internationally recognized tequila, but there are at least 80 different regional names in use due to their different features (Zizumbo-Villarreal and Colunga-GarcíaMarín, 2008). In the region of the Colima volcanoes, Vargas-Ponce et al. (2007) documented 24 morphologically distinct cultivars of *A. angustifolia* and other species that have not been taxonomically or agronomically described.

*Agave tequilana*, which is the species used to produce tequila mezcal, appears to have originated from the highly variable *A. angustifolia* complex centered in the Colima foothills (Gentry, 1982; Valenzuela Zapata and Nabhan, 2003; Vargas-Ponce et al., 2007). Possibly due to the relaxation of prohibition laws forbidding mezcal production in the mid-1800s (Luna Zamora, 1991; Valenzuela Zapata and Nabhan, 2003), large-scale commercial cultivation of *A. tequilana* for mezcal distillation gravitated to the Amatitan-Tequila Valley in central Jalisco (Bruman, 1941, 2000; Walton, 1977; Limon, 2000). Tequila production began in earnest in the mid- to late 1800s with the production of what was known as vino mezcal de Tequila (Luna Zamora, 1991; Valenzuela Zapata and Nabhan, 2003; Gaytan, 2008). The prevalence of well-drained volcanic soils in the Amatitan-Tequila Valley provided an ideal location for mezcal production (Lewis-Kenedi et al., 2005). *Agave* species naturally populate and perform well in such soils (cambisols and luvisols) (Valenzuela Zapata and Nabhan, 2003; Valenzuela, 2011).

Mezcal produced in the Amatitan-Tequila Valley increasingly became known as tequila in the early 1900s, particularly as the international export market developed (Gaytan, 2008). As a means of quality control and enhancing its marketability as a regionally defined product, regulations were enacted in Mexico in 1949 that restricted tequila to be distilled only from propagules of a vegetative clone of *A. tequilana, A. tequilana* var. Azul (blue agave) (Colunga-GarcíaMarín and Zizumbo-Villarreal, 2007; Bowen, 2010). Compared to other traditional cultivars used for tequila production, blue agave has a shorter maturation cycle, baking qualities more suitable for industrial processing, and greater production of shoots (Valenzuela Zapata and Nabhan, 2003; Colunga-GarcíaMarín and Zizumbo-Villarreal, 2007). A regulatory classification system used to protect intellectual property, known as “denominacion de origen” (or geographical indication) (Bowen and Valenzuela Zapata, 2009), was enacted in 1974 to legally define the geographic area in which tequila could be produced and labeled as such (Bowen, 2010). The system incorporates the idea of maintaining product quality and takes into consideration the concept of “terroir,” which recognizes the influence of human culture, values, soil type, climate, and native plant species (Bowen and Valenzuela Zapata, 2009). This regulation effectively restricts cultivation of blue agave to the states of Jalisco, Nayarit, Michoacan, Guanajuato, and Tamaulipas (Zizumbo-Villarreal and Colunga-GarcíaMarín, 2008).

Although mezcal production from some agaves is prohibited in some parts of Mexico (Burwell, 1995), tequila production continues to steadily increase. From 1995 to 2006, production of tequila in Jalisco and surrounding states doubled, increasing from 104 to 243 million L (Bowen, 2010). Tequila sales totaled $156 million in 1994 (Valenzuela Zapata and Nabhan, 2003). Currently, there are nearly 80,000 hectares of blue agave under production (Nobel, 2010). However, the prolonged period of maturation (6–10 years) makes it difficult to synchronize the production of blue agave with supply-demand cycles (Alisau, 2004; Bowen, 2010). Indeed, the tequila industry has been characterized since the 1930s by cycles of surplus and shortage due in part to the maturation cycle of *A. tequilana*, but also by conflicts in land use between agave farmers and tequila companies (Ruiz-Corral et al., 2002; Bowen and Valenzuela Zapata, 2009; Bowen and Gaytan, 2012). According to Bowen and Valenzuela Zapata (2009), the cycles of shortage and abundance mutually reinforce one another. When an abundance of agaves exist, low prices make it difficult for farmers to plant another crop of blue agave. In addition, during periods of surplus, farmers often lack the incentive to monitor for pests or diseases, leading to conditions ripe for epidemics (Dalton, 2005). Widespread mortality instigated by pests and disease leads to a shortage, which causes the price of blue agave to sharply rise (Ruiz-Corral et al., 2002). New producers then enter the market to capitalize upon the high prices, which then leads to current producers to expand their current plantations, typically into areas that have not been used for agave production (Ruiz-Corral et al., 2002). Due to the geographic indication restrictions, only vegetative clones of blue agave can be planted.

Coupled with increased demand, a shortage in blue agave in the late 1990s led to unprecedented increases in tequila prices (Ruiz-Corral et al., 2002; Valenzuela Zapata and Nabhan, 2003; Dalton, 2005). The shortage was caused by high levels of mortality induced by abnormally warm temperatures and high rainfall, and a disease epidemic caused by a diverse assemblage of pests and diseases, including *Scyphophorus acupunctatus* (agave weevil), *Pseudoccous* spp. (powdery louse), *Acentrocneme hesperiaris* (white worm), *Fusarium oxysporum*, and *Erwinia* spp. (Ruiz-Corral et al., 2002). Despite agaves exhibiting high levels of tolerance to various environmental stresses, monocultural production of agaves exposes plants to stresses not normally found in the wild. The lack of genetic diversity of the blue agave clone makes it particularly vulnerable to diseases and pests, especially when vegetative propagules of the same age class are widely planted (Valenzuela Zapata and Nabhan, 2003; Dalton, 2005). These ongoing cycles of abundance and shortage for tequila production undoubtedly will continue to lead to long-term cultural and environmental consequences (Bowen, 2010), including conditions ripe for another epidemic that could threaten the livelihood of those tied to the tequila industry.

The economic advantages provided by geographical indications are being compromised with the contemporary framework of tequila production (Bowen and Valenzuela Zapata, 2009). The current trend in shifting more control of blue agave production from small farmers to tequila companies to reduce their vulnerability to the surplus and shortage cycles appears to be exacerbating the problem. More mechanized, chemically intensive cultivation practices are replacing traditional methods used by small farmers. Gobeille et al. (2006) found that mechanized cultivation, which was heavily dependent on chemical inputs, actually contributed to lower soil organic matter and fertility, and also increased incidences and virulence of pests and pathogens compared to more traditional methods. In addition, intensive production practices also contribute to increased groundwater pollution and losses in floral and faunal biodiversity (Bowen and Gaytan, 2012).

Due to the many factors involved, there appears to be no easy solution to remedy the socio-environmental problems associated with tequila production. However, as Bowen and Valenzuela Zapata (2009) suggested, consideration needs to be given to recognizing the unique environmental and cultural factors of the region that originally led to the widespread popularity of tequila. Moreover, a paradigm shift might be necessary in redefining tequila. Instead of producing a homogenized, standardized product, incentives should be provided to commercially produce traditional (or artisanal) types of tequila, unique to certain regions of Jalisco and other parts of Mexico (Valenzuela Zapata and Gaytan, 2012). As such, priority should be given to conservation of increasingly marginalized traditional *A. tequilana* cultivars and landraces, several of which are on the verge of disappearing (Valenzuela Zapata and Gaytan, 2012). Such germplasm could also serve as a genetic repository for plant breeders (Valenzuela Zapata and Nabhan, 2003). Cross-pollination of these “heirloom” variants of *A. tequilana* could provide much-needed resistance to various abiotic and biotic stresses. Traditional tillage techniques, including stone terracing, could also be adopted to prevent soil erosion caused by increased expansion of agave cultivation (Gobeille et al., 2006; Bowen and Valenzuela Zapata, 2009). In addition, other traditional cultivation practices, including intercropping with maize, squash, beans, or peanuts and regular pruning of agave leaves, could help reduce the risks of pest and disease epidemics. However, the benefits of intercropping with C_3_ or C_4_ plants may be mitigated by warmer and drier conditions.

Unfortunately, the challenges that have beset tequila production will likely be realized with production of other mezcal types. Geographical indications were established for mezcal in 1994 and bacanora in 2000 (Bowen and Valenzuela Zapata, 2009). As is the case for tequila, the formal recognition process required for various mezcal products to be granted geographical indication status presents an ironic dilemma. In the process of endowing legal protections that ensure high-quality production, it works against efforts to preserve methods associated with their traditional production (Bowen and Gaytan, 2012). Also, the unrelenting cycles of shortage and surplus of blue agave will likely hold claim to other mezcal types protected by geographic indications. Moreover, the conversion to a monoculture form of production for these various agaves carries its own share of challenges. And the vagueness associated with the generic term of “mezcal” may make it difficult to regulate the harvest of agaves under cultivation and in the wild.

Some level of regulation, however, is needed to prevent overharvesting of wild-grown agaves for mezcal production (Burwell, 1995; Delgado-Lemus et al., 2014a,b). Unlike in the past where only mature agaves with numerous vegetative offsets were harvested, increased demand for mezcal could lead to more non-selective and potentially damaging harvests (Valenzuela Zapata and Nabhan, 2003; Delgado-Lemus et al., 2014a,b). Such destructive harvesting could also negatively impact agave pollinators. Up to one million wild agave plants have been estimated to be annually harvested for mezcal production in Mexico (Nabhan, 1985; Nabhan and Fleming, 1993).

Aspects of the traditional management of mezcal production could provide some solutions to problems increasingly experienced in the production of blue agave for tequila (Zizumbo-Villarreal et al., 2013). Historically, agaves were grown based on the unique edaphic and ecological conditions of the planting sites (Valenzuela Zapata and Nabhan, 2003). For example, the Mayan people intercropped agaves with beans on rocky soils (García-Mendoza, 1998). Such approaches need to be revisited. Moreover, efforts need to be made to evaluate the socio-environmental impacts of intellectual property measures related to agave cultivation. Although geographic indication regulations may provide a short-term economic boon, it may be at the cost of the environmental and cultural resources that led to the initial creation of the products.

#### Agave as a sweetener

Within the past 25 years, a growing market has emerged for another product generated from agave heads, agave syrup, which is also known as agave nectar (Garcia-Pedraza et al., 2009; Montanez Soto et al., 2011; Willems and Low, 2012). The sweetener has been marketed as a bottled product to use as a healthy alternative to table sugar, honey, and maple syrup (Willems and Low, 2012). The syrup is also being incorporated into cereals and granola bars (Zamora-Gasga et al., 2014), and as a specialized flavor sweetener for coffee (Tecklenburg, 2014). The market for agave syrup appears to be strong. Although data are limited, sales for agave syrup continue to rise. In 2007, agave syrup accounted for 15% of Wholesome Sweetener's sales in 2007, a 12% rise from the year before (Carr, 2008). Another company, Madhava Honey, had $6 million in sales of agave syrup in 2007, up from $2.9 million in 2006. The alternative sweeteners market in the U.S., which includes agave syrup, has been predicted to reach $1.4 billion by 2015 (Freedonia Group, 2011).

With the exception of some additional filtration steps, many of the initial steps used to generate tequila are essentially the same for agave syrup production (Ávila-Fernández et al., 2009; Willems and Low, 2012). The juice harvested from agave heads is subjected to acidic hydrolysis, filtered, and then vacuum evaporated to generate fructose syrup (Willems and Low, 2012; Zamora-Gasga et al., 2014). Considering that yields of blue agave often exceed that demanded for tequila production, annual losses can reach 200,000 tons of agave heads (Zamora-Gasga et al., 2014). Some of the surplus has been successfully exploited by the production of agave syrup. Indeed, nearly 10% of the harvest of blue agave and *A. salmiana* in Mexico has been estimated to be used in the production of agave syrup, with the remainder going toward tequila and mezcal production (Willems and Low, 2012). Given the volatility of the tequila and mezcal markets, having an additional market for agave products provides producers with the ability to diversify to ensure stable revenue streams.

In many circles, the appeal of agave syrup is its glycemic index (10–27), which is considerably lower than that of sucrose (68) and honey (55) (Foster-Powell et al., 2002; Willems and Low, 2012; Corrales Escobosa et al., 2014). This is largely attributable to its carbohydrate pool consisting of up to 90% fructose (Figlewicz et al., 2009). The high fructose content of agave syrup leads it to be notably sweeter than many other commercially available syrups, which consist mostly of glucose or sucrose (Willems and Low, 2012). Consequently, less agave syrup can be used to achieve a comparable amount of sweetness, leading it to be marketed as a sweetener that can result in less calorie intake. Such an approach, however, has not been immune to criticism.

Considerable controversy exists as to whether agave syrup should be considered as a healthier alternative to table sugar and other sugars. Syrup advocates suggest that its relatively low glycemic index makes it a suitable sweetener for diabetics because it causes a relatively lower spike in blood sugar than table sugar or honey (Carr, 2008). However, there are other factors to consider. Jones (2012) reported that glycemic index values are highly variable, which is due to the content and type of sugars, ingredients, and macronutrients that vary across food products. In addition, the glycemic index does not accurately represent an individual's diet, how the food was processed and cooked, or the amount eaten (Jones, 2012; Wolever, 2012). Moreover, the glycemic index should not be used as a sole metric to determine the health impacts about a given food and associated diet (Wolever, 2012). It needs to be used in conjunction with other nutritional parameters. Besides, consumers may be led to erringly think that the lower glycemic index will allow them to consume more than they would of other sweeteners.

Research in recent years has shown that overconsumption of fructose can lead to the buildup of fat in the liver, which is linked to insulin resistance and cardiovascular disease (Bray et al., 2004), and other toxic effects (Lustig et al., 2012). Stanhope et al. (2011) reported that individuals who consumed a quarter of their daily calorie requirement as high fructose corn syrup, which is 55% fructose and 45% glucose, had higher triglyceride and cholesterol levels than those who consumed pure fructose. This is, however, beyond what most people regularly consume on a daily basis. And it should be recognized that fructose is never consumed alone; it is eaten with glucose in normal diets (Hamblin, 2014).

The key issues at hand may be how agave syrup is advertised and the amount consumed. Little information exists regarding the long-term impacts on human health of consuming foods or beverages with high concentrations of fructose (Van Buul et al., 2014). Given the unknowns, people should strive to be moderate in their consumption of energy-dense foodstuffs, including agave syrup. In addition, independent of the sugar source, a calorie is a calorie as it relates to changes in body fatness (Te Morenga et al., 2013). Consumption of sugars in agave syrup appears to have the same impact on human weight loss as other sugars (Te Morenga et al., 2013). However, agave syrup should not be considered any more natural than high-fructose corn syrup or fruit-juice concentrate. And although companies have the right to market agave syrup as a sweetener, they may not be justified in suggesting it is a healthier alternative or more natural than sucrose or other widely consumed sweeteners. Making strong claims for or against the use of agave syrup should be avoided, simply because more needs to be learned regarding the impacts of fructose on human nutrition and metabolism.

### Agave as a source for bioenergy

The unique traits of agaves that make them attractive as sources of fermented and distilled beverages also offer opportunities to address immediate and long-term energy needs (Table 1). Agaves show potential as bioenergy crops due to their ability to be relatively productive on nutrient poor soils in semi-arid and arid regions. CAM photosynthesis enables agaves to be highly efficient in water and nutrient use in arid and semi-arid regions (Neales et al., 1968; Neales, 1973; Nobel, 1988; Borland et al., 2009). In addition, the long history of producing agaves for mezcal, particularly tequila, has led to a maturation and refinement of the technologies involved with the distillation process (Cedeño, 1995; Iñiguez-Covarrubias et al., 2001; Cedeño Cruz, 2003; Casas, 2006), which could be capitalized upon in their development as bioenergy crops.

Although a suite of bioenergy crops appear to be ecologically suitable for different parts of North America, such as maize (*Zea mays*) (Hill et al., 2006), switchgrass (*Panicum virgatum*) (McLaughlin and Kszos, 2005), and *Miscanthus* (Stewart et al., 2009), limited natural resources have made it difficult to turn such recommendations into reality in areas beyond the U.S. Midwest (Vanloocke et al., 2010). Also, considerable controversy surrounds the use of farmland for bioenergy crop production due to the competition it could entail with food production (Tilman et al., 2009). Agaves can be cultivated on marginal lands. Some have argued that production on such lands would not divert resources for production of conventional food crops (Tilman et al., 2009; Somerville et al., 2010). Indeed, worldwide agave cultivation occupies more than 500,000 ha, most of which is semi-arid land (Nobel et al., 2002). Moreover, marginal lands are increasing throughout the world (Kendall and Pimentel, 1994), which could allow for the turning of environmental challenges into sustainable opportunities.

Some agaves in Mexico have been documented to be highly productive (Nobel, 1991). Without supplemental irrigation, but under conditions of relatively high rainfall (771 mm yr^−1^), productivity of *A. mapisaga, A. salmiana*, and *A. tequilana* averaged 25–26 Mg ha^−1^ yr^−1^ in west-central Mexico (Nobel, 1991). When intensively managed and irrigated, yields of *A. mapisaga* and *A. salmiana* were 38 and 42 Mg ha^−1^ yr^−1^, respectively (Nobel et al., 1992). Likewise, yields of *A. fourcryodes*, which is grown for fiber, exceeded 15 Mg ha^−1^ yr^−1^ in the Yucatan Peninsula of Mexico, an area characterized by abundant precipitation (981 mm yr^−1^). However, the shallow and porous soils of the region effectively render drought-like conditions typically found in more arid regions. Under more limited rainfall conditions (320 mm yr^−1^) in relatively dry central Mexico, *A. salmiana* yielded 10 Mg ha^−1^ yr^−1^ (Nobel and Meyer, 1985), which is within range of maize yields under intensive cultivation in the Midwestern U.S. (Dohleman and Long, 2009). Agaves respond well to well-watered and fertile conditions, but can still be reasonably productive when resources are more limited. However, as suggested by Lüttge (2004, 2010), these high yields may primarily be due to C_3_ photosynthesis induced by supplemental irrigation, and not CAM photosynthesis. Moreover, the CAM photosynthetic pathway may be more of a strategy that enables plants to survive prolonged periods of drought rather than one that leads to high biomass productivity.

According to Davis et al. (2011), CAM photosynthesis provides advantages in using agaves as energy crops due to their low amounts of lignin, long considered a barrier to synthesis of lignocellulosic fuels (Somerville, 2007). In addition, CAM plants typically have relatively high amounts of soluble non-structural carbohydrates, which translate into less energy required for conversion to fuels (Borland et al., 2009). Also, in the process of producing alcohol from agave heads, pulpy residues, vinasse, and bagasse, often accumulate after the distillation process. In tequila and mezcal production in Mexico, nearly 750,000 metric tons of bagasse are produced each year (Núñez et al., 2011). Such residues could be exploited for generation of fuel or even combustible biomass to generate electricity (Hernández-Salas et al., 2009; Rendon-Salcido et al., 2009; Davis et al., 2011; Núñez et al., 2011; Simpson et al., 2011).

Using agaves, particularly *A. tequilana*, as a bioenergy crop could help resolve the problems associated with the shortage and surplus cycles that have challenged the tequila industry. Yan et al. (2011) suggested that having an alternative market to siphon off surplus harvests could provide a buffer to farmers to the financially crippling downswings in demand in the market. They attributed the surplus and shortage cycles of blue agave production to the difficulty in predicting demand several years after a crop is established. While partly true, pests and diseases, which decimate agaves in monoculture, appear to be more of the primary drivers of the cycles. The need to economize production through monoculture systems will repeatedly lead to agave crops becoming highly vulnerable to pests and pathogens. Moreover, agave ethanol production will still be subject to economic unknowns due to it competing with other sources of ethanol. *Agave* production for ethanol in U.S. and other countries will not be spared immunity from these challenges. Also, other agaves produced for bioenergy besides *A. tequilana* will not have alternative markets to protect them from the surplus and shortage cycles.

Other large hurdles will also still need to be crossed before agave production can be scaled up for its use as a bioenergy crop. With the exception of some large plantations where *A. tequilana* is cultivated for tequila production, field establishment of most cultivated agaves is primarily done manually due to the absence of mechanization in planting, cultivation, and harvesting (Bahre and Bradbury, 1980; Valenzuela Zapata and Nabhan, 2003; Gobeille et al., 2006; Valenzuela, 2011; Valenzuela Zapata and Gaytan, 2012). Some tequila companies have developed in-house machinery to increase mechanization of agave planting and harvesting (Yan et al., 2011). The general lack, however, of mechanization in Mexico, which is partly due to cheap labor, hilly terrain, and rocky soils (Gobeille et al., 2006), currently prevents widespread production of agaves in areas where labor expenses are costly. Moreover, the need to vegetatively propagate offsets of cultivars reduces the overall efficiency of agave production relative to seed-established crops, such as *Miscanthus* (Christian et al., 2005; Jørgensen, 2011). In addition to a year needed for propagating and establishing offsets of highly productive agaves, they generally require 5–8 years of growth before harvest (Núñez et al., 2011; Valenzuela, 2011). Plants are harvested individually just prior to inflorescence induction, which is when the heads reach optimal levels of size and sugar content after the sixth year. Núñez et al. (2011) reported that an *A. tequilana* field often requires up to 2 years to be fully harvested due to the amount of manual labor involved in harvesting individual agave heads. After harvesting, which is staggered due to the non-uniform growth of agaves, fields are generally left fallow for at least a year (Núñez et al., 2011). Despite agaves requiring minimal irrigation due to their physiology, many blue agave producers invest considerable resources and time for inorganic fertilizer application, chemical weed control, and pest and pathogen management in order to increase uniformity in growth and yield (Núñez et al., 2011; Valenzuela, 2011). Such an approach is likely with other agaves grown for bioenergy.

Production of agaves as bioenergy crops will likely involve increased mechanization and resource inputs, but questions remain if the ends justify the means. Part of the appeal of energy crops is for them to reduce societal dependence on petroleum and to also mitigate CO_2_ emissions. While agaves could be used as alternative sources of energy, intensive management will be required. They are not the panacea that many are seeking. The high water-use efficiency of agaves is partly due to their inherently slow growth. Risks abound for farmers due to the need to set aside land for a crop that will not be ready to harvest in 6–10 years. Moreover, the lack of uniformity in agave growth will make it difficult to recoup losses encumbered during establishment due to staggered harvests. The non-uniform nature of agave growth also demands significant time, labor, and other resources to replant where mature plants have been sporadically harvested.

Likewise, the monocarpic habit of agaves presents another set of challenges. Unlike some candidate energy crops, such as *Miscanthus* and switchgrass, which persist for several years due to their perennial, polycarpic habit (Lewandowski et al., 2003; Stewart et al., 2009), the programmed death of agave rosettes after flowering provides little room for error in determining when to harvest. Consequently, maneuvering machinery in agave plantations likely requires more energy than in a uniform corn-field, thus offsetting the physiological advantages of agaves in the net energy benefit they could provide.

The intensive labor involved with replanting of agave propagules in established fields also negatively impacts the sustainability of the production system. Unlike perennial crop systems, which have deep roots that cyclically turn over to build up soil organic matter, the shallow root systems of agaves do not substantially contribute to organic matter buildup (Nobel, 1988). Root systems of agaves comprise only 8–12% of total plant biomass. In conducting a life-cycle energy analysis of agave-derived ethanol, Yan et al. (2011) concluded that its energy output relative to its fossil energy inputs has the potential to be higher than that of ethanol produced from corn, switchgrass, and sugarcane. As they acknowledged, however, there were several unknowns in their analysis. These included the amount of energy use associated with potential large-scale planting of agaves and the actual yields of candidate species. Also, Davis et al. (2011) emphasized the need for side-by-side replicated field trials of several agaves to assess their actual productivity. Most estimates are based on growth measurements of single plants, which generally lead to overestimates of yield. Moreover, as Davis et al. (2014) emphasized, field trials will be necessary to determine the true productivity of agaves on semi-arid lands without supplemental irrigation before commercial production can be realized.

Research needs to be invested in exploring options to reduce the duration of the establishment period without dependence on fertilizers and pesticides. Molecular tools provide opportunities to manipulate time to flowering and possibly the removal of monocarpy, but the complexity of flowering genes (Weigel and Meyerowitz, 1994; Teo et al., 2014) and the presence of polyploidy (Zonneveld, 2003; Robert et al., 2008; Simpson et al., 2011) in several agaves currently present barriers to progress in these areas. Little work has been done at the molecular level to improve agaves besides *A. tequilana* (Gutiérrez-Mora et al., 2004; Simpson et al., 2011; Ruvalcaba-Ruíz et al., 2012).

### Agave as a source of fiber for ropes and fabric

Long before consideration was given to evaluating agaves as bioenergy crops, several species were harvested both in the wild and under cultivation for fiber in their leaves (Nichols et al., 2000; Rousso, 2010; King, 2011). According to Parsons and Darling (2000), agave cultivation for fiber was widespread among the large civilizations in the highlands of central Mexico prior to Spanish colonization. Agave leaves were harvested to extract fibers to make various products, such as thread for cloth textiles, rope, bags, and footwear (Evans, 1990; Parsons and Parsons, 1990). Complex infrastructure was established for agave fiber processing, as reflected in the large production areas discovered in the Aztec city-state of Otumba (Nichols et al., 2000). However, vibrant household economies also existed where families would grow agaves in house gardens to extract, spin, and weave fibers to create their own clothing (Plunket and Urunuela, 2012), and possibly also for tributes to the Aztec state (Evans, 1990). Parsons and Darling (2000) estimated that nearly 1 million people (i.e., the approximate number of people living in the Valley of Mexico in AD 1500) could have been clothed from fiber harvested from 10,000 ha of agaves. Based on their calculations, these plantations would have only used 5% of arable land in the Valley, thereby only minimally impacting food production. While textiles from agave fibers have been found in archaeological sites in central Mexico dating back to 6500 BC (Schery, 1972), the apex of pre-Columbian use of agaves by the Aztecs appears to have occurred during the Late Postclassic period (AD 1300–1519).

Although cotton was also grown to make textiles, it was limited to warmer areas in fertile soils (Nichols et al., 2000). However, agave could be grown at much higher elevations in drier soils than cotton or even maize. Such an agricultural strategy helped sustain the high level of organizational complexity found in Aztec society in a region characterized by aridity, severe winter frosts, and highly seasonal rainfall (Sauer, 1963; Nichols et al., 2000; Parsons and Darling, 2000). Soil erosion and frost-kill were minimized in the highlands of the Teotihuacan Valley through the use of complex terraced systems in the higher elevations (2400–2500 m asl), such as in the ancient village of Cihuatecpan (Evans, 1990). Species that were cultivated for fiber, but also for food and beverage, by the Aztecs included *A. americana, A. ferox, A. hookeri, A. mapisaga*, and *A. salmiana* (Gentry, 1982; Parsons and Darling, 2000).

Across the Gulf of Mexico on the Yucatan Peninsula and south into Central America, *A. fourcroydes* (henequen) was cultivated by the Maya for several hundred years for the production of fiber (Sheets, 2000; Colunga-GarcíaMarín, 2003). Details are sparse regarding how the Maya cultivated and used henequen. Much of their historical records (codices) were destroyed by the Spanish (Colunga-GarcíaMarín et al., 1996). What is known, however, is that by the time of Spanish colonization, henequen had been domesticated (Colunga-GarcíaMarín, 2003). According to Colunga-GarcíaMarín et al. (1996), the Franciscan monk, Diego de Landa observed that henequen was cultivated in gardens, and was of higher quality than wild species (possibly *A. angustifolia*). Excavations at an ancient Mayan village, Joya de Ceren, in El Salvador, which was buried under volcanic ash over 1400 years ago, corroborate with the observations of de Landa (Sheets, 2000). Several agave plants were found growing in a household garden with an area set aside for extracting fiber from harvested plants. So extensive was the plant selection of the Maya that at least seven different landraces of henequen were developed (Yaax Ki, Sac Ki, Chucum Ki, Bab Ki, Kitam Ki, Xtuk Ki, and Xix Ki) (Colunga-GarcíaMarín et al., 1996), of which only three remain in cultivation today (Sac Ki, Yaax Ki, and Kitam Ki) (Colunga-GarciaMarin and May-Pat, 1993).

Henequen is a relatively infertile species, possibly due to interactive effects between its pentaploid nature (Zonneveld, 2003) and the asynchronous development of its male and female gametophytes during and after meiosis (Piven et al., 2001). As with *A. tequilana*, henequen descended from *A. angustifolia* (Gentry, 1982; Colunga-GarcíaMarín and Maypat, 1997; Colunga-GarcíaMarín et al., 1999; Colunga-GarcíaMarín, 2003), which is a fertile hexaploid (Castorena-Sanchez et al., 1991), and has a wide native distribution throughout Mexico, including the Yucatan Peninsula (Gentry, 1982; Colunga-GarcíaMarín et al., 1996; Colunga-GarcíaMarín, 2003). The sterile nature of henequen likely was a factor in its widespread cultivation for use as fibers for clothing and cordage (Granick, 1944; Zonneveld, 2003), even by the pre-Columbian Maya. Granick (1944) observed that agave polyploids had much thicker, coarser, and succulent leaves than conspecific diploids, which likely led to their widespread commercial use, such as pentaploid *A. sisalana* and tetraploid *A. lechuguilla*.

Soon after colonization, the Spanish recognized the suitability of henequen as a fiber crop, and increased its cultivation to produce clothing, cordage, and other products for the empire and as a trade item (Colunga-GarciaMarin and May-Pat, 1993). Driven by demand for binder twine for harvesting wheat in the U.S. in the late 1800s, and for ropes for ships, henequen cultivation was scaled up in the Yucatan Peninsula (Wells, 1985; Colunga-GarcíaMarín, 2003; Evans, 2013). The primary landrace that was planted was the Sac Ki cultivar, which was preferred by the cordage industry for its high quality and relatively long fibers. The widespread planting of the Sac Ki cultivar ultimately led to the demise of several other landraces. Growth of the henequen fiber industry continued into the early 1900s with the widespread use of fiber-extracting machinery, which facilitated mass production (Andrews et al., 2012). The industry peaked between 1910 and 1914, but declined over the next several decades with many of the plantations becoming abandoned by the 1950s (Andrews et al., 2012). The initial blow to the industry occurred in 1915, with the abolition of indentured servitude during the Mexican Revolution. According to Gentry (1982), agave fiber production yields profits only if cheap manual labor is available.

Just as the unique morphology and physiology of agave complicate the commercialization of its production as a bioenergy crop, the same holds true for its use as a fiber crop. Carstensen (1986) suggests that the relatively long maturation cycle of agaves may have made it difficult for producers to respond to price increases, which could possibly lead to the same damaging supply-demand cycles that plagues *A. tequilana* production (Ruiz-Corral et al., 2002; Dalton, 2005; Bowen and Valenzuela Zapata, 2009; Bowen and Gaytan, 2012).

In addition, the increase in competition of henequen with the production of *A. sisalana*, whose fiber is commercially superior (Brockway, 1979), in the Americas and Africa, along with the advent of synthetic fiber production, also led to a weakening of the henequen industry in Mexico (Wells, 1985; Shamte, 2001; Evans, 2013). Nationalization of the industry, beginning in the 1960s, also crippled the industry (Shamte, 2001). While a shadow of its former self, however, the henequen industry persists in Mexico, and leads in production worldwide (Food and Agriculture Organization, 2009). Annual production has been around 17,000 metric tons in Mexico for the past several years (Food and Agriculture Organization, 2009). Cuba is the only other country that produces a significant amount of henequen, but its annual production averages around 1,400 metric tons.

Although not prominent as a fiber crop in Mexico, *A. sisalana* (sisal) was long cultivated in east Africa and Brazil (Osborne and Singh, 1980). Although its origins are not clearly known, it presumably originated in the state of Chiapas, Mexico (Gentry, 1982). The species was then introduced into Florida from the Yucatan Peninsula in 1836 as a potential fiber crop by a horticulturist who was a former consul to Mexico, Henry Perrine (Robinson, 1942; Klose, 1948). As a means of promoting agave fiber production in British colonies throughout the tropics, the Kew Botanic Garden Bulletin, provided, along with other agronomic information regarding the agave fiber industry in Mexico, information about sisal plants being cultivated in Florida (Brockway, 1979). A German agronomist, Richard Hindorf, capitalized upon this information and ordered 1,000 sisal bulbils in 1893 from a nursery in Florida to establish a fiber industry in Tanzania, which had been recently colonized by Germany (Glenk et al., 2007; Tenga, 2008). Underscoring the advantages and risks in vegetative propagation of agaves, only 62 of the original 1,000 bulbils survived the overseas shipment, but served as the foundational germplasm for the sisal industry in Tanzania (Kimaro et al., 1994).

Despite these setbacks, however, sisal production steadily increased up through the first half of the twentieth century in East Africa (Kimaro et al., 1994). The widespread cultivation of a high-yielding hybrid, “Hybrid 11648,” which was introduced in East Africa in 1960, helped contribute to increased productivity (Peregrine, 1969). The peak of annual production, however, climaxed in the late 1960s at nearly 230,000 metric tons in Tanzania (Kimaro et al., 1994; Sabea, 2001). It then declined to nearly 30,000 tons by the mid-1980s. Not only did global markets for sisal fiber shrink partly due to the increased demand for synthetic fibers, but also due to the nationalization of sisal plantations after Tanzania and Kenya became independent from British rule in the 1960s (Kimaro et al., 1994; Sabea, 2001; Food and Agriculture Organization, 2009). Nationalization led to wholesale changes in ownership and management of sisal production companies, which unfortunately led to mismanagement of sisal plantations and the depletion of a highly skilled workforce. The high susceptibility of Hybrid 11648 to pests and diseases, such as *Phytopothora arecae* (zebra leaf rot or bole rot), may have also played a role (Peregrine, 1969; Kimaro et al., 1994). As of 2008, global production amounted to 248,000 metric tons, but the global players have changed. Approximately 40% of the total fibers harvested in 2008 were in Brazil, which has led in sisal fiber production for the past several years (Food and Agriculture Organization, 2009). Production has also been increasing again in recent years in Tanzania (Oudshoorn, 1995; Food and Agriculture Organization, 2009).

Although decorticating machines, such as periquitas in Brazil, are widely used nowadays to extract fibers from leaves (Satyanarayana et al., 2007), many aspects of the fiber extraction process have remained unchanged for hundreds of years (Colunga-GarciaMarin and May-Pat, 1993; Rousso, 2010). Even in industrial settings, the entire process largely involves strenuous and hazardous manual labor (Gilson et al., 1962; Kimaro et al., 1994; Sabea, 2001; Kayumba et al., 2008a, 2009). Regardless of the method used, the leaves are harvested, generally by hand, and then the spine tips and side teeth are removed. The fibers are then extracted from the leaves using one of three primary methods, soaking (retting), roasting, or steaming (Rousso, 2010). All three methods are used to separate the fibers from the outer matrix of leaf material, which has been softened or weakened in the exposure to water and/or fire. However, environmental risks abound for each of the methods, both for rural artisans and for industrial-scale applications. Soaking leaves in ponds, rivers, or lakes leads to oxygen depletion in water bodies, which, coupled with the seepage of the acidic sap of the leaves into the water, has led to high levels of fish kill in many areas (Shamte, 2001; Rousso, 2010). Using fire to help extract fibers requires a significant amount of wood, which has taxed the natural resources in many areas, especially Central America, both for ancient and modern societies (Parsons and Darling, 2000; Rousso, 2010).

Even though it has been suggested that marginal lands in semi-arid climates are highly suitable for agave cultivation (Somerville et al., 2010; Davis et al., 2011), Kimaro et al. (1994) reported that commercial sisal production requires high levels of inputs, including fertilization and irrigation. In particular, soil fertility is a major limiting factor to continued sisal production in Tanzania. Sustained production of sisal on the same lands year after year depletes soil nutrient reserves, particularly calcium, to almost urecoverable levels (Kimaro et al., 1994). As with several other CAM species, calcium levels in agaves tend to be higher than in many plants (Nobel and Berry, 1985), but the reasons for this are not clearly understood (Lüttge, 2004). However, crop rotation of hybrid sisal with a legume species has been found to restore soil fertility levels (Kimaro et al., 1994).

While sisal tolerates drought, optimal rainfall for high productivity is around 1,200 mm yr^−1^ (Kimaro et al., 1994). Such responsiveness to mesic conditions has been observed in some agaves (Hartsock and Nobel, 1976; Nobel, 1991; Nobel et al., 1992), and challenges the paradigm that they can rely solely upon CAM photosynthesis to be highly productive. While agaves are certainly more adapted to semi-arid environments and could be produced more sustainably than many conventional crops, such as maize, expecting yields comparable to many high-yielding biomass crops without commensurate irrigation might not be realistic. This is, however, an area that needs to be further researched, possibly in large field trials that track diurnal and nocturnal carbon gain over several years.

Every crop has its limitations. While *Miscanthus* x*giganteus* has long been considered a potential energy crop for the U.S. Midwest, recent research has shown that it transpires copious amounts of water (Vanloocke et al., 2010), thereby mitigating its effectiveness as a sustainable source of biomass or biofuel. Both the advantages and disadvantages of agaves as crops need to be fully considered regardless of what its end uses are.

As has been mentioned, sisal production is labor intensive, but workers have also been documented to be exposed to health and safety hazards while harvesting and processing the fibers (Brockway, 1979; Sabea, 2001; Kayumba et al., 2007, 2008a,b, 2009, 2011). While some might consider this to be an issue that will eventually disappear with the overall decline of the industry, sisal fiber production has increased in recent years due to the development of new uses for sisal. The light-weight nature of the fibers plus their biodegradability have led to them being evaluated as reinforcements in polymer-based composites as alternatives to asbestos and fiberglass (Bisanda and Ansell, 1992; Filho et al., 1999; Li et al., 2005). Some have even considered them as a source of bioenergy (Oudshoorn, 1995).

One driving factor in the renewed interest of sisal fibers is the perceived low cost in their production and processing. However, prices of sisal fibers have in large measure not accounted for the aforementioned human capital costs and health risks of laborers, which are primarily located in developing nations (Brockway, 1979). While processing sisal, workers are exposed to high levels of dust, and have had high incidences of respiratory symptoms, which have led to reduced lung function (Stott, 1958; Mustafa et al., 1978; Baker et al., 1979; Zuskin et al., 1994; Kayumba et al., 2007, 2008a, 2009, 2011). If the sisal industry is to be revived, efforts need to be made to ensure safe working conditions for laborers. Research should be focused also on improving the mechanization of sisal production.

## Conclusions

Agave exhibits remarkable qualities both in the wild and under cultivation. Its evolved adaptations to hot, dry environments coupled with its diverse end products warrant its use as a model CAM crop. Equipped with the water-use efficient CAM pathway and unique morphophysiological traits, agaves benefit the overall functioning of their native ecosystems (Gentry, 1982; Lüttge, 2010; Nobel, 2010). These same traits have also led to their use by mankind for millennia as sources of food, beverage, medicine, and clothing. Indeed, in some parts of Mexico and Central America, little has changed in their use over the centuries (Rousso, 2010; Zizumbo-Villarreal et al., 2013; Correa-Ascencio et al., 2014). However, these traits that enable agaves to survive and sustain productivity in water-limited regions have led to a high degree of versatility in the services and products they provide to society in a warming world.

While there are no immediate prospects of cultivating agave as a staple food crop, avenues should be explored in using inulin-type fructans from agaves as prebiotics, and also as foodstuff incorporated into processed foods. And considering the purported nutritional benefits of pulque, more effort should be made to improve the sanitation of how it is produced to allow for more widespread commercialization. Medicinal benefits from agaves are also continuing to be discovered (Orestes Guerra et al., 2008; Ahumada-Santos et al., 2013).

Those involved in the governance and production of tequila and other forms of mezcal need to give consideration to the long-term ecological, cultural, and economic impacts of geographic indications, particularly in how to minimize the negative impacts of shortage-surplus cycles. Easement of such legislation should be considered, particularly to allow for long-established landraces to be used to produce regionally unique forms of agave spirits with traditional production methods that are inherently more sustainable than more modern mechanized approaches. The spectacular rise in consumption of agave syrup offers a potential means of mitigating the negative impacts of shortage-surplus cycles, but increased clarity is needed regarding its purported health benefits.

Opportunities also exist to harness the starch-rich heads and succulent leaves of agaves into ethanol fuel (Cáceres-Farfán et al., 2008; Somerville et al., 2010; Davis et al., 2011; Escamilla-Trevino, 2012). However, the physiology of agave constrains the ability for it to be considered a carbon-neutral bioenergy crop without significant improvements in the current energy-intensive methods used to grow and harvest agaves. Research particularly needs to address the constraints associated with its long maturation cycles. Similar limitations are associated with the use of agave as a fiber crop. The environmental and human health costs associated with agave fiber production also need to be assessed before efforts are made at reviving the industry.

CAM photosynthesis has conferred the ability for agaves to establish and be moderately productive in resource-limited environments, but its limitations need to be understood both within ecological and economic contexts. As Lüttge (2010) emphasized, CAM photosynthesis is a strategy for survival and not for high productivity. Depending on the end product, they can be considered low-impact, sustainable crops, but in many cases, they are equivalent to many conventional crops in terms of resource use. As such, agaves warrant use for several commercial applications, but their restrictions need to be better recognized. Overall, however, agaves in natural and cultivated settings provide a means to help resolve ecological and economic disconnect between natural and human systems, particularly in resource-limited environments.

### Conflict of interest statement

The author declares that the research was conducted in the absence of any commercial or financial relationships that could be construed as a potential conflict of interest.
